# HMRM: A Hybrid Motion and Region-Fused Mamba Network for Micro-Expression Recognition

**DOI:** 10.3390/s25247672

**Published:** 2025-12-18

**Authors:** Zhe Guo, Yi Liu, Rui Luo, Jiayi Liu, Lan Wei

**Affiliations:** School of Electronics and Information, Northwestern Polytechnical University, Xi’an 710179, China; liuyi_3276@mail.nwpu.edu.cn (Y.L.); rui_l@mail.nwpu.edu.cn (R.L.); liujiayiqqq@mail.nwpu.edu.cn (J.L.); weilan@mail.nwpu.edu.cn (L.W.)

**Keywords:** micro-expression recognition, optical flow, Mamba, region fusion, intelligent visual sensing

## Abstract

Micro-expression recognition (MER), as an important branch of intelligent visual sensing, enables the analysis of subtle facial movements for applications in emotion understanding, human–computer interaction and security monitoring. However, existing methods struggle to capture fine-grained spatiotemporal dynamics under limited data and computational resources, making them difficult to deploy in real-world sensing systems. To address this limitation, we propose HMRM, a hybrid motion and region-fused Mamba network designed for efficient and accurate MER. HMRM enhances motion representation through a hybrid feature augmentation module that integrates gated recurrent unit (GRU)-attention optical flow estimation with a regional MotionMix enhancement strategy to increase motion diversity. Furthermore, it employs a grained Mamba encoder to achieve lightweight and effective long-range temporal modeling. Additionally, a regions feature fusion strategy is introduced to strengthen the representation of localized expression dynamics. Experiments on multiple MER benchmark datasets demonstrate that HMRM achieves state-of-the-art performance with strong generalization and low computational cost, highlighting its potential for integration into compact, real-time visual sensing and emotion analysis systems.

## 1. Introduction

Micro-expressions (MEs) are brief, involuntary facial movements triggered by attempts to conceal genuine emotions, typically lasting less than 0.5 s [1]. Despite their subtlety, MEs reveal critical emotional cues and hold value in high-stakes domains such as criminal investigation, clinical diagnosis, and public safety [2,3]. In the context of intelligent visual sensing, these fleeting expressions provide vital signals for emotion-aware perception and decision making [4,5]. Recent advances in artificial intelligence and computer vision have spurred interest in automatic micro-expression recognition (MER), enabling sensing system to access latent human affective states in a non-intrusive manner [6]. However, the transient, low-intensity, and low-saliency nature of MEs poses significant challenges for modeling their fine-grained spatiotemporal dynamics within practical sensing environments [7].

Traditional MER methods primarily rely on hand-crafted features such as texture descriptors and optical flow to capture subtle facial dynamics [8,9]. While these approaches offer basic automation, they often fail to accurately model key muscle movements and are highly sensitive to noise and illumination changes, limiting their robustness. With the rise of deep learning, Convolutional Neural Networks (CNNs) [10] and CNN-based multi-stream architectures [11,12,13] have become prevalent in MER, leveraging multimodal inputs (e.g., RGB, optical flow) to enhance dynamic feature extraction. However, due to their inherently local receptive fields, CNNs struggle to capture long-range dependencies, creating performance bottlenecks [14]. To address this, recent research has explored Vision Transformers (ViTs) [15,16,17] and Graph Neural Networks (GNNs) [18,19], which improve global context modeling and inter-regional relationship learning. In parallel, learning paradigms such as ensemble methods [20] and self-supervised contrastive learning [21] have been introduced to further boost performance. Nevertheless, these models often rely on complex architectures and large-scale training, making them resource-intensive and prone to overfitting in limited-data scenarios. Striking a balance between fine-grained dynamic modeling and lightweight deployment remains an open challenge in MER [2].

To address the above challenges, we propose HMRM, a Hybrid Motion and Region-fused Mamba network tailored for efficient and accurate MER in resource-constrained settings. HMRM is designed to enhance motion perception, regional representation, and sequence modeling while maintaining a compact architecture. Specifically, we propose a Hybrid Motion Feature Augmentation (HMFA) module that integrates a Gated Recurrent Unit (GRU)-attention optical flow estimation mechanism with a MotionMix enhancement strategy to amplify subtle motion cues and improve data diversity. We further design a Grained Mamba Encoder, built upon the linear-time Mamba framework, to achieve efficient multi-scale spatiotemporal encoding while capturing long-range dependencies with minimal computational overhead. Additionally, we develop a Regions Feature Fusion Strategy (RFFS) that partitions the face into semantically meaningful regions and applies cross-scale interaction to enhance regional dynamics and reduce redundancy. By jointly leveraging motion-guided augmentation, state-aware sequence modeling, and region-level fusion, HMRM improves the model’s sensitivity to micro-expression dynamics while ensuring efficient deployment. Extensive experiments on multiple public MER benchmarks demonstrate that HMRM achieves state-of-the-art performance with robust generalization and low computational cost. It is well suited for deployment in intelligent visual sensing systems and real-world emotion-aware applications.

Our main contributions are summarized as follows:

- We propose a Hybrid Motion Feature Augmentation Module, incorporating GRU-attention optical flow estimation and MotionMix enhancement, to jointly enhance the modeling of subtle facial dynamics and increase the generalizability of training data.
- We introduce a Grained Mamba Encoder, leveraging the state space modeling capabilities of Mamba for lightweight and efficient spatiotemporal encoding. In addition, we design a Regions Feature Fusion Strategy to strengthen the representation of critical facial regions and cross-regional interactions.
- We present a novel, lightweight MER framework, HMRM, which achieves a favorable trade-off between accuracy and efficiency, and outperforms existing methods across several benchmark datasets.

## 2. Related Work

### 2.1. MER Methods Based on Hand-Crafted Features

Early MER methods based on hand-crafted features exhibit unique theoretical and practical value. These approaches primarily rely on domain knowledge to manually design spatiotemporal feature descriptors that capture subtle facial dynamics. Among them, texture-based methods such as Completed Local Binary Patterns from Three Orthogonal Planes (CLBP-TOP) [8] and its improved variant Spatiotemporal Local Binary Pattern with Integral Projection (STLBP-IP) [22] are representative. By encoding information from the horizontal, vertical, and temporal dimensions, they marked the initial attempts toward automated MER. However, such methods predominantly focus on static texture cues and are inherently limited in modeling motion amplitudes and muscle movement trajectories, the key attributes of MEs. Furthermore, they exhibit high sensitivity to illumination changes, limiting their robustness in real-world conditions.

To address these shortcomings, motion-based approaches employing optical flow have been introduced to capture facial movement in terms of direction, magnitude, and velocity. Representative methods include Main Directional Mean Optical-flow (MDMO) [9] and Bi- Weighted Oriented Optical Flow (Bi-WOOF) [23], which enhance recognition performance via region-of-interest (ROI) partitioning and weighting schemes. Nonetheless, optical flow-based methods also suffer from the loss of fine-grained details and vulnerability to noise. Despite notable progress, hand-crafted methods generally suffer from limited scalability, high computational complexity, and poor adaptability across datasets, which hinders their effectiveness in complex, real-world scenarios [8].

### 2.2. MER Methods Based on Deep Learning

With the success of deep learning in computer vision, MER has benefited from the powerful feature learning and representation capabilities of data-driven models. Early deep learning-based MER approaches primarily utilized CNNs [10], applying transfer learning or end-to-end training strategies. However, due to the limited size of available MER datasets and the transient nature of micro-expressions, single-frame modeling often failed to capture sufficient temporal dynamics. To improve spatiotemporal representation, multi-stream architectures [24] have been proposed, which integrate optical flow and temporal cues into the learning process. This trend continued with the development of three- and four-stream networks [11,12,13], which further incorporate motion patterns and domain-specific priors to boost recognition accuracy. Despite these advances, the intrinsic locality of CNNs hinders their ability to model long-range dependencies, which are essential for understanding subtle MEs changes.

More recently, Transformer-based models have been explored for MER due to their global attention mechanisms. Vision Transformer (ViT)-based frameworks [25] and their derivatives have been employed to capture holistic spatiotemporal correlations, with studies introducing Transformer-based feature fusion [26,27,28], optical flow-guided attention [29], and hierarchical region-aware modeling strategies [30]. In parallel, GNNs have been leveraged to model structured motion dependencies across facial regions [31].

Nevertheless, most methods still rely heavily on optical flow, which remains a bottleneck. Conventional optical flow algorithms [32] often fail to capture the subtle motion patterns in MER, while more accurate alternatives are computationally expensive and impractical for real-time applications [33]. To address these challenges, we propose a novel lightweight MER framework that integrates motion-guided enhancement with fine-grained regional modeling. By combining hybrid motion perception and Mamba-based encoding, our method captures subtle facial dynamics more effectively while maintaining high efficiency.

### 2.3. Mamba

While many recent MER frameworks attempt to improve accuracy by increasing model complexity, the quadratic computational cost of Transformer-based attention mechanisms severely limits their scalability and real-time applicability. The recently proposed Mamba framework [34] provides a promising alternative. Based on the selective State Space Model (SSM), Mamba integrates a continuous-time dynamical system with discrete-time recurrence, enabling efficient and expressive sequence modeling. Its selective scan mechanism adaptively controls the flow of critical information while suppressing irrelevant signals, enhancing both modeling robustness and computational efficiency. Mamba has shown strong performance across tasks requiring fine-grained spatial and temporal modeling, including vein recognition [35], medical image analysis [36], and skin lesion segmentation [37]. Its ability to focus on key regions and filter redundant signals makes it particularly suitable for MER, where motion cues are both subtle and localized.

Current MER methods struggle to balance recognition accuracy with model efficiency. To address this, we propose a lightweight, motion-aware framework that integrates Mamba for fine-grained spatiotemporal encoding. By leveraging Mamba’s efficient inference capabilities, our approach aims to enhance motion detail extraction while reducing computational cost and deployment complexity, making it more suitable for real-world micro-expression analysis.

## 3. Method

**Figure 1:**
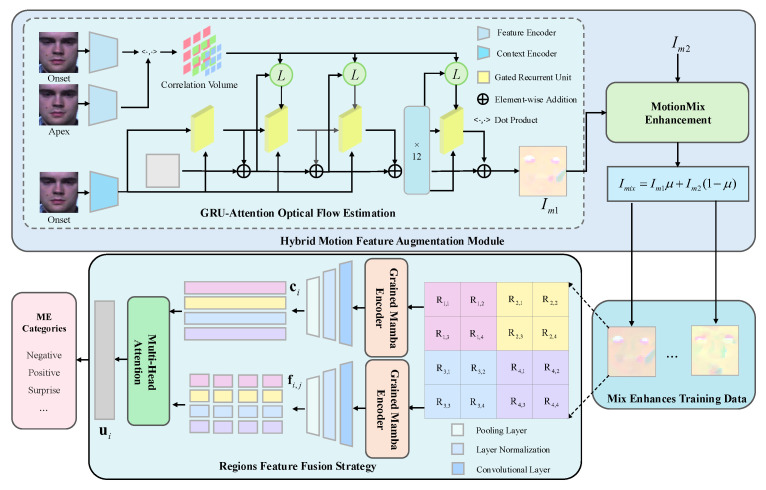
Overview of the proposed HMRM framework. The model integrates a Hybrid Motion Feature Augmentation module, a Grained Mamba Encoder, and a Regions Feature Fusion Strategy to jointly enhance motion perception, regional representation, and temporal modeling for efficient and accurate micro-expression recognition.

We propose HMRM, a lightweight end-to-end framework for MER that effectively balances recognition performance and computational efficiency. HMRM is designed to robustly model fine-grained facial dynamics by integrating motion-guided feature enhancement mechanism with region-aware representation learning in a unified architecture. By enabling efficient and accurate perception of subtle facial motions, HMRM provides a promising foundation for intelligent visual sensing systems to achieve reliable emotion analysis in real-world environments. As illustrated in Figure 1, the framework comprises three key components: the Hybrid Motion Feature Augmentation (HMFA) module, a Grained Mamba Encoder, and the Regions Feature Fusion Strategy (RFFS). Together, these modules enable robust and efficient modeling of subtle facial dynamics. Given a pair of onset frame $I_{o}$ and apex frame $I_{a}$, they are first passed through a shared Feature Encoder to produce their corresponding down-sampled feature maps $F_{o}$ and $F_{a}$, respectively. Additionally, $I_{o}$ is also processed by a separate Context Encoder to extract the context feature, computed as a 4D Correlation Volume via dot products between all feature pairs from $F_{o}$ and $F_{a}$, followed by multi-scale pooling. The resulting features are fed into the GRU-Attention Optical Flow Estimation (GRU-AOFE) module. This module iteratively updates a hidden state and optical flow estimate via a Gated Recurrent Unit (GRU) combined with a self-attention mechanism, producing a dense optical flow map $I_{m1}$ that emphasizes subtle motion while suppressing noise. Next, the MotionMix Enhancement module selects a secondary flow map $I_{m2}$ from the training set and performs landmark-guided patch extraction around key regions (eyes, mouth). Local patches are swapped and linearly blended with a ratio μ to generate a synthetic optical flow map $I_{mix}$. This yields an augmented set of flow maps (original and synthetic), improving motion diversity without introducing artifacts. Each flow map is spatially divided into 4 coarse-grained regions, with each further subdivided into 2×2 fine-grained patches $R_{i,j}$. These are input to the Grained Mamba Encoder, which leverages the efficiency of linear-time State Space Models (SSMs) for parallel region-level sequence modeling. It outputs coarse region vectors $c_{i}$ and fine-grained vectors $f_{i,j}$, enabling efficient multi-scale encoding. Finally, the Region Feature Fusion Strategy (RFFS) aggregates both levels of features using a Multi-Head Self-Attention mechanism. Cross-scale interactions yield fused vectors $u_{i}$, which are concatenated and passed through a fully connected layer for classification. RFFS promotes region-aware dynamic modeling while maintaining a lightweight architecture. Through the synergy of HMFA, Grained Mamba Encoder, and RFFS, HMRM achieves efficient and robust MER with fine-grained motion perception and strong generalization.

### 3.1. Hybrid Motion Feature Augmentation Module

Compared to macro-expression recognition, MER requires capturing subtle and localized facial muscle movements. However, existing methods based on frame sequences or optical flow either incur high computational costs or fail to effectively capture fine-grained motion details. To address these limitations, we propose the Hybrid Motion Feature Augmentation (HMFA) module, which comprises two components: a GRU-AOFE mechanism that enhances the quality of motion representation, and a MotionMix Enhancement strategy that augments the training set with diverse yet label-consistent motion patterns. This dual design improves both the discriminative power and generalizability of motion features for MER.

#### 3.1.1. GRU-Attention Optical Flow Estimation

The GRU-AOFE module estimates optical flow between the onset and apex frames to capture the spatiotemporal dynamics of MEs. Inspired by RAFT [38], we adopt a lightweight GRU-based architecture to enable efficient deployment on resource-constrained devices. To suppress noise and enhance motion feature extraction in key facial regions, we integrate a self-attention mechanism within the GRU.

For the onset frame $I_{o}$ and apex frame $I_{a}$ in a ME, we estimate a dense motion field $\left(f^{o},f^{a}\right)$ that maps each pixel location (u,v) from the onset frame to the apex frame, resulting in an optical flow map $I_{m}=\left(u+f^{o}\left(u,v\right),v+f^{a}\left(u,v\right)\right)$. The optical flow estimation part has two input pipelines. On the one hand, a ResNet is used to perform down-sampled feature encoding on $I_{o}$ and $I_{a}$, mapping them to feature maps $F_{o}$ and $F_{a}$ at 1/8 resolution. On the other hand, a Context Encoder ψ(x) with an identical structure is used only on the onset frame $I_{o}$ for down-sampled feature extraction. Subsequently, a 4D Correlation Volume *C* is computed for the feature maps from the first pipeline, expressed by the formula:

(1) $$C\left(i,j\right)=\langle F_{o}\left(i\right),F_{a}\left(j\right)\rangle \in R^{H\times W\times H\times W}$$

where *i* and *j* represent the pixel indices of $F_{o}$ and $F_{a}$; *H* and *W* represent the 1/8 height and 1/8 width of the input frames, respectively. To capture subtle movements while preserving high-resolution information, we construct a 4-level multi-scale feature pyramid by applying pooling operations with kernel sizes of 1, 2, 4, and 8 on the last two dimensions of the Correlation Volume, formulated as

(2) $$C_{l}\left(i,j\right)=\mathrm{AveragePooling}\left(C_{l-1}\left(i,j\right)\right)$$

**Figure 2:**
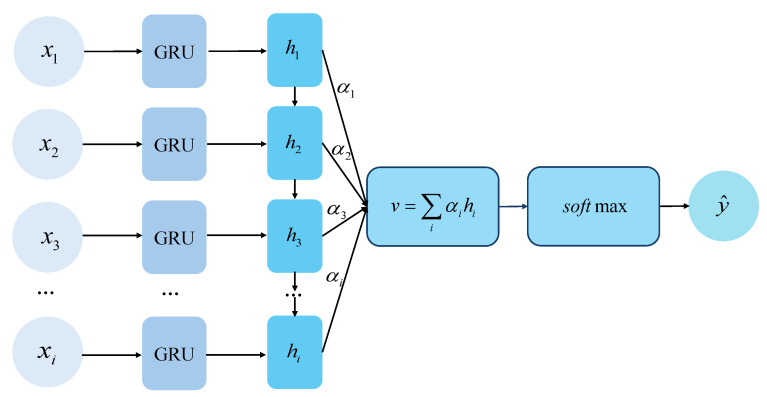
Computation process of the GRU-Attention Optical Flow Estimation module. The module integrates gated recurrent updates with an attention mechanism to refine motion representations, emphasizing subtle facial movements while suppressing irrelevant background information.

This encodes pixel-wise similarity across scales and serves as input to a GRU-based recurrent update module, which iteratively refines the optical flow. Since the resulting flow features are high-dimensional and may include background noise, we embed a self-attention mechanism within the GRU. This enables the model to focus on key facial regions while suppressing irrelevant information. The overall computation process is illustrated in Figure 2, where $x_{i}$ is the current input and $h_{i}$ is the previous hidden state. The attention weights between each time step are calculated as follows:

(3) $$\alpha_{i}=\frac{\exp(e_{i})}{\sum_{j}\exp\left(e_{j}\right)}$$

(4) $$e_{i}=\mathrm{score}\left(h_{i},s\right)=v_{a}^{T}\tanh\left(W_{a}h_{i}+U_{a}s\right)$$

where *s* is the state of the current decoder, and additive attention is used to compute the attention score $e_{i}$, with $W_{a}$ and $U_{a}$ being learnable weights. $v_{a}\in R^{d}$ is a learnable vector that projects the combined features into a scalar attention score. Finally, the estimated optical flow output is obtained as

(5) $$v=\sum_{i}\alpha_{i}h_{i}$$

(6) $$\hat{y}=\mathrm{softmax}\left(W_{o}v+b_{o}\right)$$

where *v* is the context vector, $W_{o}$ and $b_{o}$ are parameters of the output layer, and $\hat{y}$ is the final estimated optical flow. By computing attention weights, the model identifies the importance of each region, enabling focused feature extraction from key MEs areas and producing an optical flow map $I_{m}$ that effectively captures their dynamic patterns.

#### 3.1.2. MotionMix Enhancement

To enrich motion diversity under limited-data conditions, we propose MotionMix enhancement, a lightweight augmentation strategy targeting key facial regions. It synthesizes new optical flow samples by exchanging the eye and mouth regions between two maps with the same class label. This preserves label consistency while introducing local motion variations, guided by the dynamic features from the GRU-AOFE module. The process can be formally represented as

(7) $$I_{mix}=I_{m1}\mu +I_{m2}\left(1-\mu \right)$$

where $I_{m1}$ and $I_{m2}$ denote two optical flow maps with the same class label, and $I_{mix}$ represents the mixed flow map. The regions μ correspond to the eye and mouth areas, which are localized using facial landmark detection. During mixing, these regions are swapped between the two maps to generate $I_{mix}$, while the class label $label_{mix}$ is inherited from the original samples. Since the modification is limited to local motion without altering the overall expression semantics, label consistency is maintained.

By synthesizing diverse local motion combinations, MotionMix Enhancement enriches the training set and introduces greater variation in expression patterns. This improves the model’s ability to generalize and enhances its sensitivity to subtle regional motion cues critical for MER.

### 3.2. Grained Mamba Encoder

To enable efficient temporal modeling and better contextual encoding for MEs sequences, we propose the Grained Mamba Encoder, built upon the Mamba framework, an efficient sequence modeling method based on selective SSMs [34]. Mamba integrates continuous-time dynamical systems with discrete-time recursion, offering strong capability in capturing long-range dependencies.

Formally, Mamba maps an input sequence u(t) to hidden states x(t), producing output y(t) via continuous-time SSM dynamics. Its discrete-time formulation using Zero-Order Hold (ZOH) is given by

(8) $$y_{k}=C\left(Ax_{k}+Bu_{k}\right)+Du_{k}$$

where *A*, *B*, *C*, and *D* are input-dependent dynamic parameters. This selective SSM design allows Mamba to adaptively focus on salient temporal patterns, demonstrating superior performance in various sequence modeling tasks.

**Figure 3:**
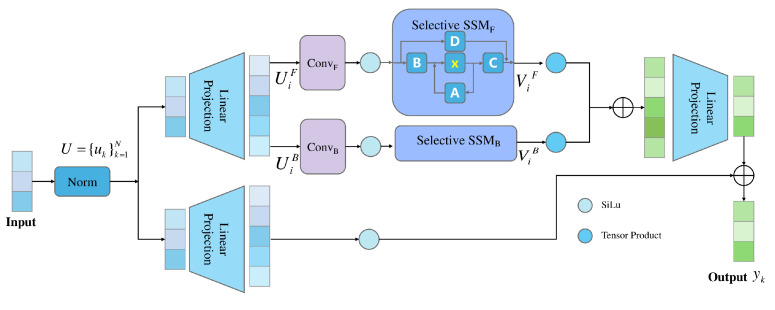
Architecture of the Grained Mamba Encoder. The encoder performs bidirectional state space modeling on multi-scale motion features, enabling efficient temporal context learning and fine-grained encoding of localized facial dynamics.

To adapt Mamba for MER, we introduce a multi-scale, local-aware mechanism that preserves Mamba’s global modeling strengths while improving sensitivity to localized motion dynamics. As shown in Figure 3, the input optical flow map is first partitioned into patches and embedded as a token sequence $U={\left\{u_{k}\right\}}_{k=1}^{N}$, where each $u_{k}$ represents the feature of a patch. After normalization and linear projection, we obtain the forward sequence:

(9) $$U_{i}^{F}=\mathrm{Linear}\left(\mathrm{Norm}\left(U\right)\right)$$

To capture bidirectional temporal dependencies and alleviate the loss of local information caused by independent token processing, we apply convolutional operations to both the forward and backward projected sequences. Specifically, the forward and backward features are computed as

(10) $$V_{i}^{F}=M_{i}^{F}\left(\sigma \left(U_{i}^{F}⊗W_{i}^{F}\right)\right)$$

(11) $$V_{i}^{B}=M_{i}^{B}\left(\sigma \left(U_{i}^{B}⊗W_{i}^{B}\right)\right)$$

where $W_{i}^{F}$ and $W_{i}^{B}$ are learnable 1D convolution kernels, σ(·) represents the SiLU activation function, and $M_{i}^{F}$ and $M_{i}^{B}$ represent the computation processes of the forward and backward SSM modules, respectively.

The outputs from both directions are then fused using a gating mechanism and combined with the original input through a residual connection to produce the final output $y_{k}$. The bidirectional structure enriches temporal context modeling, while the adjustable convolutional kernel sizes enable multi-granularity feature encoding, allowing the encoder to adaptively focus on distinct motion scales within different regions of the ME sequence.

### 3.3. Regions Feature Fusion Strategy

To enhance the representation of localized facial dynamics and efficiently capture inter-regional dependencies, we propose a Regions Feature Fusion Strategy (RFFS) that integrates physiological region partitioning with multi-scale feature modeling. This strategy not only improves sensitivity to subtle motion patterns but also reduces redundant computation, exhibiting strong scalability and practical applicability.

As illustrated in Algorithm 1, given an input high-quality optical flow map F, we first partition it into four primary regions $R_{i}$ (top-left, top-right, bottom-left, bottom-right), corresponding to the periocular and perioral zones—two key regions activated during micro-expressions. This coarse-grained division helps suppress irrelevant background noise and guides the model to focus on semantically salient areas, in line with the functional coordination of facial muscles.
**Algorithm 1** Region Feature Fusion Strategy**Require:** Optical flow image F, Number of core regions R=4, Number of subdivisions per region S=4, Feature encoder E**Ensure:** Fused feature representation Z  1:Segment F into four core regions $\left\{R_{i}|i=1,\ldots ,4\right\}$;  2:**for** each core region $R_{i}$ **do**  3:   Subdivide $R_{i}$ into *S* sub-regions $\left\{R_{i,j}|j=1,\ldots ,S\right\}$;  4:   **for** each sub-region $R_{i,j}$ **do**  5:    Extract fine-grained feature $f_{i,j}\leftarrow E\left(R_{i,j}\right)$;  6:   **end for**  7:   Extract coarse-grained feature $c_{i}\leftarrow E\left(R_{i}\right)$;  8:**end for**  9:Gather all fine-grained features $\left\{f_{i,j}\right\}$ and coarse-grained features $\left\{c_{i}\right\}$;10:Fuse multi-grained features: $Z\leftarrow \mathrm{Fusion}\left(\left\{f_{i,j}\right\},\left\{c_{i}\right\}\right)$;11:**return** Z

For each sub-region $R_{i,j}$ and its corresponding parent region $R_{i}$, we apply the Grained Mamba Encoder to extract fine-grained features $f_{i,j}$ and coarse-grained features $c_{i}$, respectively. These features are then fed into a multi-head attention fusion module that performs cross-granularity and cross-regional interaction, yielding the final fused representation Z. This coarse-to-fine and integrative fusion paradigm strengthens the model’s ability to capture subtle muscle deformations while maintaining robustness under real-world conditions such as illumination changes and partial occlusion.

### 3.4. Loss Function

To fully exploit the benefits of multi-scale spatial feature modeling in MER, we design a Multi-Scale Weighted Cross-Entropy (MS-WCE) loss that introduces a scale-aware weighting mechanism into the conventional cross-entropy formulation. This mechanism adaptively emphasizes discriminative features across different spatial scales, enhancing the model’s sensitivity to dynamic micro-expression patterns while improving convergence efficiency. The designed loss is defined as

(12) $$L_{\mathrm{MS}-\mathrm{WCE}}=\sum_{i,s}w_{i,s}\left(-\log\left(p_{t,s}^{i}\right)\right)$$

where *i* denotes the sample index, *s* indicates the feature scale (including both core and sub-region levels), $w_{i,s}$ is the learnable or pre-defined importance weight for the scale *s*, and $p_{t,s}^{i}$ is the predicted probability for the target class. Following a focal-style reweighting scheme, the probability term is expressed as

(13) $$p_{t,s}^{i}=p_{c,s}^{y_{i}}{\left(1-p_{c,s}\right)}^{1-y_{i}}$$

where $p_{c,s}$ is the confidence predicted by the model for class *c* on scale *s*, and $y_{i}$ is the ground-truth label. This formulation helps balance hard and easy samples across scales, improving learning stability and generalization.

By aligning with the multi-scale representation extracted via the Grained Mamba Encoder and the Region Feature Fusion Strategy, our MS-WCE loss ensures that both coarse and fine-grained facial dynamics are optimally supervised, leading to improved recognition performance under varying expression intensities and spatial granularities.

## 4. Experiments

### 4.1. Datasets and Evaluation Metrics

#### 4.1.1. Datasets

We evaluate our method on three widely used MER benchmark datasets: CASME II [39], SMIC-HS [40], and SAMM [41]. To assess the generalization and robustness of our proposed HMRM framework across heterogeneous sources, we further construct a composite dataset by merging the three.

The CASME II, constructed by the Institute of Psychology, Chinese Academy of Sciences, contains 247 spontaneous MEs sequences captured at a high frame rate of 200 fps, with a resolution of 280 × 340. It uses a three-stage (onset–apex–offset) frame annotation method and covers seven basic emotions, such as happiness, disgust, surprise, repression, sadness, fear, and others [39].

The SMIC-HS contains 164 samples collected at 100 fps with a resolution of 640 × 480 through a “punishment threat” experimental paradigm, focusing on capturing the dynamic features of MEs in naturalistic settings. While the video resolution is identical to CASME II, the effective resolution of the facial region in SMIC-HS is reported to be lower, which can impact the visibility of subtle expression features [40].

The SAMM offers 159 high-resolution samples (2040 × 1088), covering a diverse multi-ethnic population and is annotated with eight emotion dimensions [41].

For the composite dataset, all sequences are first normalized and aligned using Dlib’s 68-point facial landmark detection. Following the Composite Database Evaluation (CDE) protocol from MEGC 2019 [42], emotion categories are unified into three classes: negative (e.g., anger, disgust, fear), positive (happiness), and surprise. The resulting dataset comprises 442 sequences from 68 subjects: 250 negative, 109 positive, and 83 surprise samples. Our MER network adopts an onset–apex frame architecture. As SMIC-HS lacks explicit onset/apex annotations, we follow the approximation method proposed in [43]. For facial region localization, we employ MTCNN [44] to extract landmark coordinates required by our region-aware processing modules.

#### 4.1.2. Evaluation Metrics

To address the challenges of inter-subject variability and class imbalance in MER tasks, we adopt the Leave-One-Subject-Out (LOSO) cross-validation strategy. In each iteration, one subject’s data is used for testing while the remaining subjects’ data are used for training. This process is repeated for all subjects, effectively removing subject-specific bias and maximizing data utilization under small-sample constraints (*N* <500).

We employ two class-agnostic evaluation metrics: unweighted F1 score (UF1) and the unweighted average recall (UAR) [45], which jointly assess classification performance across all emotion categories. The UF1 is calculated as the macro-average of class-wise F1 scores:

(14) $$UF1=\frac{1}{\left|C\right|}\sum_{c\in C}\frac{2\cdot Precision_{c}\cdot Recall_{c}}{Precision_{c}+Recall_{c}},$$

where *C* is the set of emotion classes, and $Precision_{c}$, $Recall_{c}$ are computed as

(15) $$Precision_{c}=\frac{TP_{c}}{TP_{c}+FP_{c}},\;Recall_{c}=\frac{TP_{c}}{TP_{c}+FN_{c}}.$$

The UAR is defined as the average recall across all classes:

(16) $$UAR=\frac{1}{\left|C\right|}\sum_{c\in C}\frac{TP_{c}}{TP_{c}+FN_{c}}.$$

where $TP_{c}$, $FP_{c}$, and $FN_{c}$ denote the true positives, false positives, and false negatives for class *c*, respectively.

Together, UF1 and UAR provide a robust and unbiased evaluation under class imbalance, avoiding the dominance of majority classes. Additionally, we report the number of parameters and FLOPs to evaluate model efficiency, including memory cost, computational complexity, and deployment feasibility in Section 4.3.

### 4.2. Implementation Details

The GRU-AOFE module is pre-trained on FlyingChairs [46] and FlyingThings [47] for 100k iterations with a batch size of 10 to learn general motion representations, which are subsequently applied to estimate optical flow for micro-expression sequences, as described in Section 3.1. The resulting flow maps are further enhanced through the MotionMix strategy to increase temporal diversity and improve the robustness of motion representation. The Grained Mamba Encoder, detailed in Section 3.1.1, is configured with an embedding dimension of 192, a network depth of 4, and a state dimension of 16 to capture fine-grained spatiotemporal dependencies. The Regions Feature Fusion Strategy introduced in Section 3.3 adopts a 7 × 7 local attention window to effectively balance local feature precision with global contextual perception. Model optimization is performed using the AdamW optimizer with an initial learning rate of 0.0005 and a weight decay of 0.01 over 1000 epochs. All experiments are conducted on an Ubuntu 20.04.1 platform equipped with an NVIDIA RTX 4090 GPU and an Intel Xeon Gold 6271C CPU, ensuring high computational throughput and real-time inference capability. This experimental configuration validates both the effectiveness of the optical flow estimation and the overall efficiency of the proposed HMRM framework.

### 4.3. Comparison with State-of-the-Art MER Methods

To comprehensively evaluate the performance of the proposed HMRM framework, we compare it against a diverse set of representative MER methods spanning traditional, deep learning, and hybrid paradigms. We include classical hand-crafted feature-based approaches such as CLBP-TOP [8] and Bi-WOOF [23], as well as deep CNN models including GoogLeNet [48], VGG16 [49], and CapsuleNet [43], which learn expressive features directly from MEs data. Optical flow-based methods like OFF-ApexNet [24] are also considered due to their capacity to model motion dynamics. To capture spatiotemporal dependencies more effectively, we incorporate temporal modeling and attention-enhanced approaches such as STSTNet [11] and GEME [50]. We also include MobileViT [51], a lightweight Transformer-based model that demonstrates strong performance under constrained computational resources. Finally, we compare HMRM with recent state-of-the-art (SOTA) methods, including FRL-DGT [28], HTNet [30], and MFDAN [29], which represent the current frontier in MER research. All comparison methods have been rigorously validated on public benchmarks such as CASME II, SMIC-HS, and SAMM, ensuring the fairness and credibility of our evaluation. This comprehensive comparison enables an objective assessment of HMRM’s performance, highlighting both its strengths and limitations relative to established and cutting-edge methods.

As shown in Table 1, the proposed HMRM method outperforms all existing baselines across all evaluation metrics, with the exception of the SMIC-HS dataset, where it ranks second. On the composite dataset, HMRM achieves a UF1 of 0.8788 and a UAR of 0.8906, surpassing the previous state-of-the-art method. Notably, on the high-resolution SAMM dataset, HMRM improves performance by over 9% compared to the best existing method. This improvement is attributed to the rich motion detail present in SAMM, which allows our method to fully exploit its strengths in optical flow estimation and long-range temporal modeling via the Grained Mamba Encoder. The encoder’s ability to capture fine-grained motion patterns while suppressing redundant information contributes to this superior performance. In contrast, on the lower-resolution SMIC-HS dataset, HMRM’s advantage diminishes. The limited spatial detail restricts the benefits of optical flow modeling, and our relatively simple attention design falls short of competing with more elaborate attention-based architectures. In addition to the primary metrics, UF1 and UAR, Table 2 presents the Accuracy (ACC) results of the HMRM method on the evaluation datasets, offering a complementary perspective on performance. It is important to note that while Accuracy provides a standard measure, we primarily focus on UF1 and UAR for micro-expression recognition due to their robustness in handling the severe data imbalance commonly found in this domain. The high ACC values achieved across all datasets further validate the effectiveness and generalizability of our proposed HMRM method.

**Figure 4:**
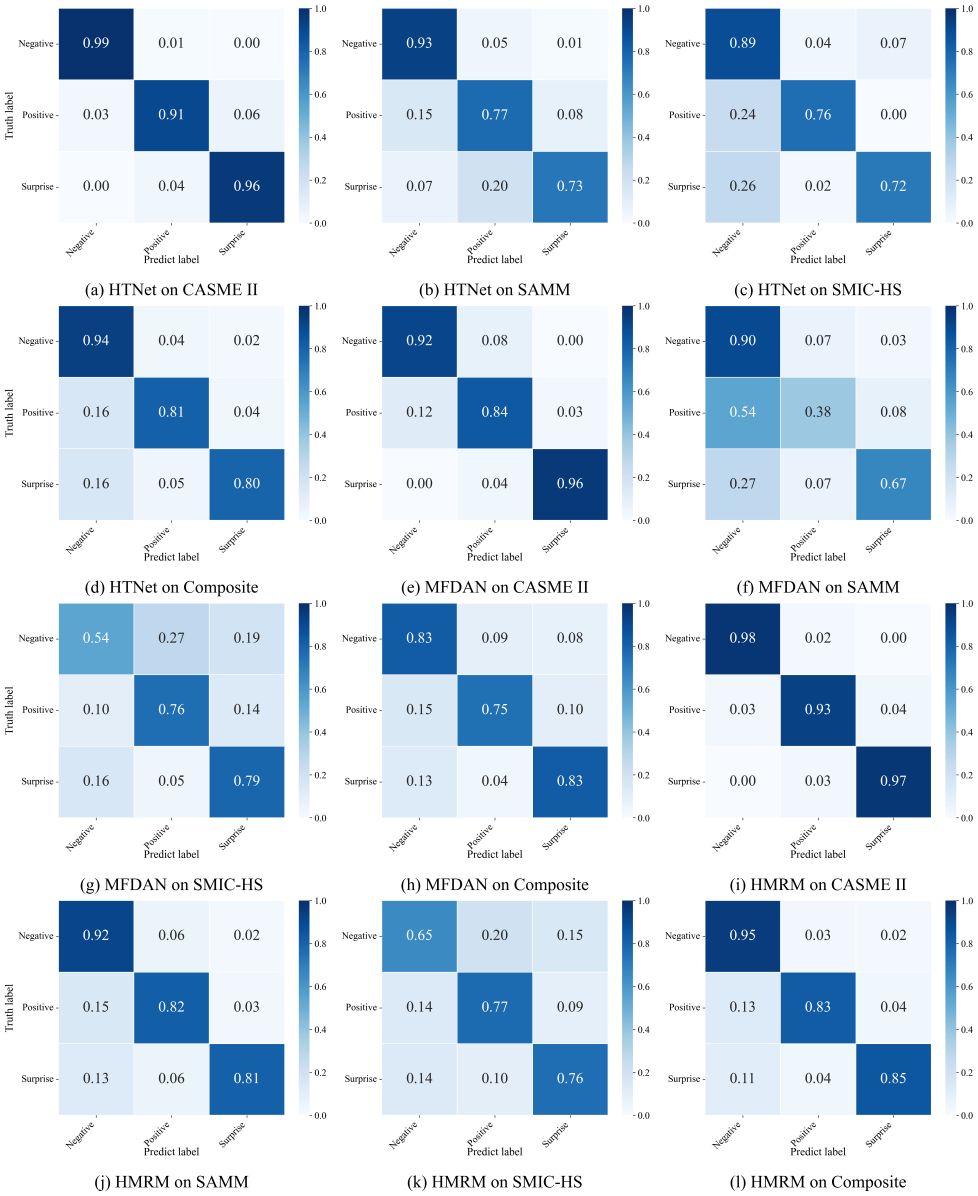
Confusion Matrices for Micro-Expression Recognition, comparing HTNet, MFDAN, and HMRM across the CASME II, SAMM, SMIC-HS, and Composite datasets.

To gain deeper insight into the classification behavior of different models, Figure 4 illustrates the confusion matrices of evaluation dataset, and composite datasets. HMRM achieves higher true-positive ratios across all emotion categories, particularly for the Surprise class, where conventional models often suffer from high inter-class confusion. Compared with HTNet and MFDAN, HMRM exhibits lower misclassification rates between Negative and Positive expressions, demonstrating the advantage of its fine-grained regional modeling and motion-guided augmentation in discriminating subtle affective cues. On the high-resolution SAMM datasets, nearly all diagonal values exceed 0.8, indicating that HMRM effectively captures localized muscle activations with minimal false recognition. In contrast, the performance gap on SMIC-HS remains moderate due to its lower spatial resolution, which limits optical flow fidelity. Overall, the confusion matrices confirm that HMRM yields more balanced and discriminative predictions across emotion categories, validating the robustness and generalization of its hybrid motion-region fusion strategy.

**Figure 5:**
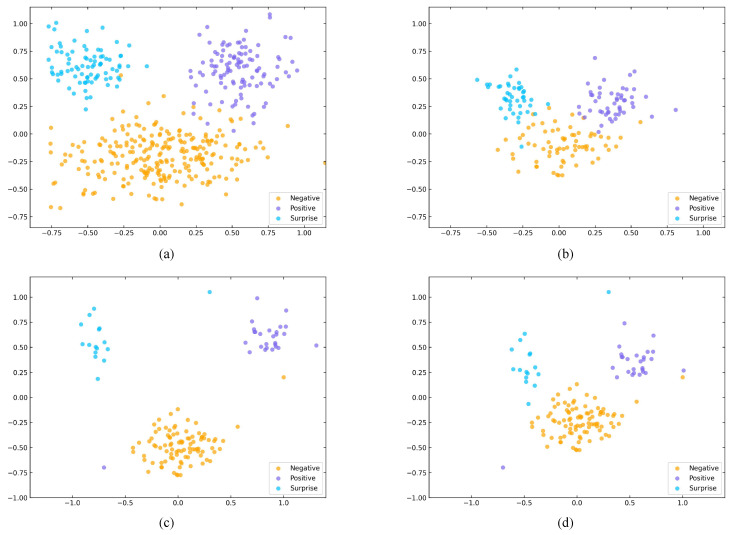
Comparison of the feature distributions generated by HMRM on the evaluation dataset. (**a**) Composite dataset; (**b**) SMIC-HS dataset; (**c**) SAMM dataset; (**d**) CASMEII dataset.

To further investigate the representational behavior of HMRM, we visualize the feature distributions of the three MEs classes on each individual dataset (CASME II, SMIC-HS, and SAMM) as well as the composite dataset, as shown in Figure 5. The results reveal that features on the SAMM dataset form more compact clusters, whereas those on SMIC-HS appear more scattered. This observation aligns with the input resolution of each dataset: higher-resolution inputs offer richer motion cues, facilitating better feature separability. Correspondingly, HMRM demonstrates superior classification performance on CASME II and SAMM, indicating its strong capacity to extract discriminative features from high-quality or feature-dense inputs.

**Figure 6:**
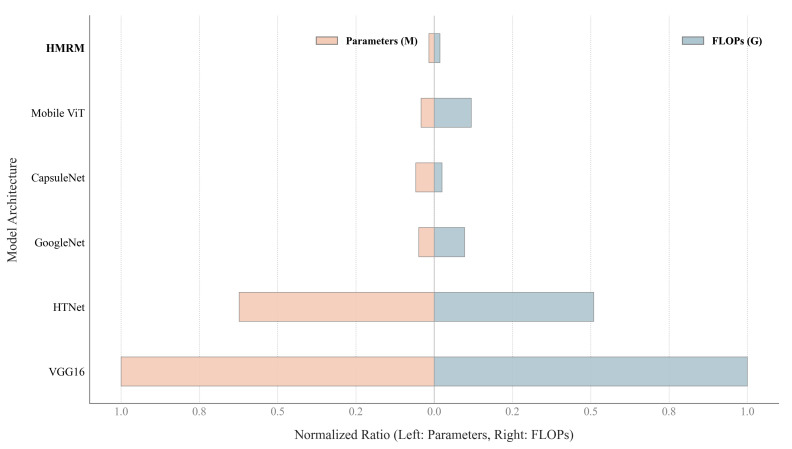
Comparison of model complexity across architectures. The vertical axis shows the model architectures evaluated, including VGG16, HTNet, GoogleNet, CapsuleNet, Mobile ViT, and HMRM. The horizontal axis represents the normalized ratio, where the left side corresponds to the number of parameters in millions and the right side corresponds to the number of floating-point operations in billions.

In terms of model lightweighting, we used the same optical flow maps as input and conducted feature extraction using five common models as well as our method. The comparison of model parameter counts and FLOPs is shown in Figure 6. Our method achieves an effective trade-off between model size and recognition accuracy. Specifically, it reduces parameter count by nearly 50% compared to models with similar performance, while maintaining competitive accuracy. This compact design effectively lowers hardware requirements, enabling efficient integration of HMRM into embedded visual sensing devices and facilitating real-time emotion recognition in practical sensing systems.

### 4.4. Ablation Study

To assess the individual contributions of the core components in HMRM, we conduct ablation experiments on the CASME II dataset [39], focusing on three modules: (1) GRU-AOFE, (2) MotionMix Enhancement, and (3) RFFS. The CASME II dataset contains 88 Negative, 32 Positive, and 25 Surprise samples, providing a balanced evaluation for module-level analysis. The detailed results are summarized in Table 3. In the ablation setting, four models are defined: M1 refers to replacing GRU-AOFE with the traditional TV-L1 optical flow [37], M2 removes MotionMix data augmentation, M3 excludes the RFFS module, and M4 represents the complete version of the proposed HMRM. For fair comparison, when evaluating the influence of a specific module, all other components and hyperparameters are kept consistent.

#### 4.4.1. GRU-Attention Optical Flow Estimation

In model M1, the GRU-AOFE module is ablated and replaced with the classical TV-L1 optical flow algorithm, allowing us to examine the effect of attention-based optical flow estimation within the HMRM framework. As shown in the first row of Table 3, the baseline performance using TV-L1 yields a UF1 of 0.9032 and UAR of 0.9164, which is approximately 5% lower than the results obtained with GRU-AOFE. This performance gap underscores the critical role of precise optical flow estimation in MER tasks. Micro-expression recognition heavily relies on capturing fine-grained motion features in localized facial regions. The GRU-AOFE module, enhanced by an attention mechanism, models temporal dependencies more effectively, improves sensitivity to subtle muscle movements, and suppresses redundant information from non-expressive regions, resulting in superior feature representation compared to traditional optical flow approaches.

#### 4.4.2. MotionMix Enhancement

In model M2, the MotionMix Enhancement module is removed to assess the influence of synthetic motion diversity on model generalization. The second row of Table 3 presents the results without incorporating the MotionMix Enhancement module. The resulting UF1 and UAR are 0.9207 and 0.9324, respectively, indicating a noticeable drop in overall performance, particularly for underrepresented emotion classes. Upon integrating MotionMix, the UF1 improves to 0.9561 (+3.54%) and UAR to 0.9588 (+2.64%). Specifically, the F1 scores for Negative, Positive, and Surprise emotions increase by 4.1%, 3.8%, and 8.9% respectively, with the Surprise class improving from 0.8213 to 0.8947. These results confirm that MotionMix enhances the model’s capacity to extract dynamic features by leveraging temporal diversity through multi-frame optical flow fusion. It demonstrates strong generalization, particularly in class-imbalanced scenarios.

#### 4.4.3. Regions Feature Fusion Strategy

Model M3 excludes the Regions Feature Fusion Strategy to evaluate the role of regional feature aggregation in improving spatial discrimination. As shown in Table 3, the UF1 and UAR drop to 0.9174 and 0.9253, respectively. This performance degradation indicates that the model lacks spatial discrimination and robustness when RFFS is excluded. After incorporating RFFS, both UF1 and UAR reach 0.9561 and 0.9588, demonstrating the module’s substantial benefit. RFFS enhances local motion sensitivity by segmenting the optical flow map into four core facial regions and sixteen sub-regions, followed by region-wise feature extraction via the Grained Mamba Encoder. This hierarchical spatial decomposition and cross-region fusion allows for more accurate modeling of micro-expression dynamics, especially transient and localized motion patterns.

The fourth row of Table 3 corresponds to model M4, which integrates all the proposed modules, representing the complete HMRM framework. This configuration achieves the highest UF1 and UAR scores, validating the complementary contributions of GRU-AOFE, MotionMix Enhancement, and RFFS to overall model performance.

#### 4.4.4. Grained Mamba Encoder Hyperparameter Analysis

**Figure 7:**
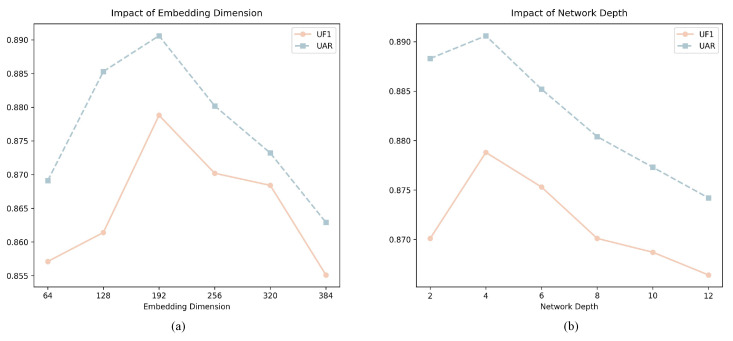
Impact of the Grained Mamba Encoder’s hyperparameters on MER performance. (**a**) Embedding dimension; (**b**) Network depth.

To further explore the performance sensitivity of the Grained Mamba Encoder, we conduct an additional ablation study on the combined dataset, focusing on two hyperparameters: embedding dimension and network depth. Specifically, we vary the embedding dimension from 64 to 384 in steps of 64, and the depth from 2 to 12 in steps of 2. All other parameters remain fixed. As shown in Figure 7, the best performance is achieved when the embedding dimension is set to 192 and the depth to 4. This configuration strikes a balance between representation capacity and computational complexity.

## 5. Limitations

Despite the strong performance of HMRM, several limitations remain that should be acknowledged to guide future research.

Dataset imbalance: Micro-expression datasets inherently exhibit severe class imbalance, which may influence both model optimization and evaluation reliability. Following the MEGC 2019 protocol, all emotion labels are unified into three categories: negative, positive, and surprise. The class distributions are as follows: CASME II (60.7% negative, 22.1% positive, 17.2% surprise), SMIC-HS (42.7% negative, 31.1% positive, 26.2% surprise), SAMM (69.2% negative, 19.5% positive, 11.3% surprise), and the composite dataset (56.6% negative, 24.7% positive, 18.8% surprise). This imbalance may lead the model to favor majority classes and under-represent the subtle dynamics of minority categories such as positive and surprise expressions. While our hybrid motion region fusion strategy improves generalization under sparse data conditions, achieving fully balanced performance across classes remains challenging.

Limited dataset scale: MER datasets contain only a few hundred labeled samples, which restricts the learning of high capacity models and may limit the robustness of long-range temporal modeling. Although HMRM incorporates lightweight Mamba based encoding to mitigate overfitting, the scarcity of annotated micro-expressions remains a fundamental bottleneck for both supervised and hybrid training paradigms.

Potential dataset bias: Existing MER benchmarks are recorded under controlled laboratory environments with constrained illumination, head pose, and background conditions. As a result, the generalizability of HMRM to real-world sensing scenarios, where expressions may be occluded, partially visible, or embedded in cluttered scenes, cannot be fully guaranteed. Additional evaluation on in the wild micro-expression like datasets or cross-domain settings would provide deeper insights into model robustness.

Computational trade-offs: Although HMRM is designed to be lightweight, its hybrid motion augmentation module (GRU-attention optical flow and MotionMix) introduces additional computations compared with extremely compact real-time architectures. Deploying the model on edge devices may still require further pruning or quantization.

These limitations highlight multiple opportunities for future exploration, including class imbalance aware training strategies, larger-scale MER data collection, domain adaptation methods, and further optimization toward hardware friendly deployment.

## 6. Discussion

To better understand the limitations of the proposed method and explore potential directions for improvement, we analyze a set of challenging cases from the composite dataset, comparing HMRM with two state-of-the-art models: HTNet [30] and MFDAN [29], as shown in Table 4.

Although HMRM achieves top overall performance, certain samples remain difficult to classify accurately. In some instances, expressions exhibit mixed emotional cues, for example, positive expressions in the lower face and negative cues in the upper face, which introduces ambiguity and leads to misclassification. Additionally, some samples feature low-intensity or indistinct micro-expressions, particularly around the eyes and mouth, making them inherently harder to recognize. Another observed challenge stems from label subjectivity, as ground-truth annotations can vary based on the annotator’s interpretation, introducing noise into the learning process. To address these issues, future work will explore uncertainty-aware learning strategies, such as soft-label modeling and probabilistic decision boundaries, to mitigate the impact of annotation ambiguity and enhance robustness when handling ambiguous or borderline samples.

## 7. Conclusions

This work addresses the longstanding challenge of balancing recognition accuracy and computational efficiency in MER by proposing a lightweight, end-to-end framework HMRM. The framework integrates a GRU-Attention-based optical flow estimation module with a MotionMix Enhancement strategy, effectively enhancing the spatiotemporal motion feature representation of facial motion signal. In parallel, the incorporation of a Grained Mamba Encoder and a multi-scale regional feature fusion strategy enables precise modeling of subtle facial dynamics while maintaining computational efficiency, making it well suited for intelligent visual sensing and emotion-aware perception systems.

Extensive experiments conducted on three benchmark MER datasets, CASME II, SMIC-HS, and SAMM, demonstrate that HMRM consistently outperforms existing SOTA methods on most evaluation metrics. Notably, on the SAMM dataset, HMRM achieves a UF1 of 0.8909 and UAR of 0.9017, confirming its superiority in terms of recognition performance and robustness. Despite its strengths, HMRM has several limitations. First, its performance degrades on low-resolution datasets such as SMIC-HS, revealing limitations in the current feature extraction design for coarse-grained visual inputs. Second, the limited size and class imbalance of available MER datasets may hinder the model’s generalization to unconstrained real-world scenarios. Additionally, while HMRM focuses on the eye and mouth regions, it does not explicitly model global facial muscle dynamics or subtle head movements, both of which could provide complementary cues for more robust sensing based recognition.

Future research will aim to further enhance the performance, generalization, and efficiency of HMRM in real-world sensor-driven and vision-based emotion-sensing applications. We plan to optimize the feature extraction module to improve robustness under low-resolution and low-quality video conditions, and investigate super-resolution-based preprocessing and resolution invariant feature learning strategies to address performance degradation on challenging datasets. In addition, we will explore self-supervised, weakly supervised, and domain adaptation paradigms to effectively leverage large-scale unlabeled facial video data and improve generalization across diverse environments. Enhancing annotation consistency will also be an important direction, where semi-supervised or label refinement approaches may help mitigate the influence of subjective labeling. Furthermore, we intend to develop more comprehensive dynamic modeling strategies that integrate global and pose invariant facial dynamics, enabling more accurate and holistic multimodal perception. Finally, to facilitate deployment in resource-limited sensing devices and edge computing systems, we will investigate model compression and adaptive inference techniques for lightweight optimization and real-time performance. 

## Figures and Tables

**Table 1:** Performance comparison with state-of-the-art methods on CASME II, SMIC-HS, SAMM, and the composite dataset under the LOSO protocol. Bold indicates the best result, and underlined denotes the second-best.

Method	Composite		SMIC-HS		SAMM		CASME II	
	UF1	UAR	UF1	UAR	UF1	UAR	UF1	UAR
CLBP-TOP [8]	0.5882	0.5785	0.2000	0.5280	0.3954	0.4102	0.7026	0.7429
Bi-WOOF [23]	0.6296	0.6277	0.5727	0.5829	0.5211	0.5139	0.7805	0.8026
GoogLeNet [48]	0.5573	0.6049	0.5123	0.5511	0.5124	0.5992	0.5989	0.6414
VGG16 [49]	0.6425	0.6516	0.5800	0.5964	0.4870	0.4793	0.8166	0.8202
CapsuleNet [43]	0.6520	0.6506	0.5820	0.5877	0.6209	0.5989	0.7068	0.7018
OFF-ApexNet [24]	0.7196	0.7096	0.6817	0.6695	0.5409	0.5392	0.8764	0.8681
STSTNet [11]	0.7353	0.7605	0.6801	0.7013	0.6588	0.6810	0.8382	0.8686
GEME [50]	0.7402	0.7501	0.6294	0.6572	0.6870	0.6541	0.8402	0.8510
FGRL-AUF [27]	0.7914	0.7933	0.7192	0.7215	0.7751	0.7890	0.8798	0.8710
MobileViT [51]	0.6981	0.7318	0.7356	0.7141	0.6781	0.7428	0.6997	0.7215
FRL-DGT [28]	0.8120	0.8110	0.7430	0.7490	0.7720	0.7580	0.9190	0.9030
HTNet [30]	0.8603	0.8475	**0.8049**	**0.7905**	0.8131	0.8124	0.9532	0.9516
MFDAN [29]	0.8453	0.8688	0.6815	0.7043	0.7871	0.8196	0.9134	0.9326
HMRM (Ours)	**0.8788**	**0.8906**	0.7491	0.7759	**0.8909**	**0.9017**	**0.9561**	**0.9588**

**Table 2:** Accuracy of the proposed HMRM method on the evaluation dataset.

Method	Composite	SMIC-HS	SAMM	CASME II
HMRM (ours)	86.7%	80.5%	85.7%	93.8%

**Table 3:** Ablation study of different modules in the proposed HMRM method. Bold indicates the best result.

Models	GRU-AOFE	MotionMix Enhancement	RFFS	CASME II	
				UF1	UAR
M1	×	√	√	0.9032	0.9164
M2	√	×	√	0.9207	0.9324
M3	√	√	×	0.9174	0.9253
M4	√	√	√	**0.9561**	**0.9588**

**Table 4:** Comparison of recognition results of HMRM with SOTA methods on challenging cases.

Labels	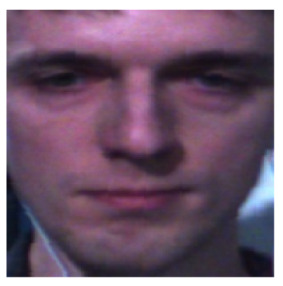	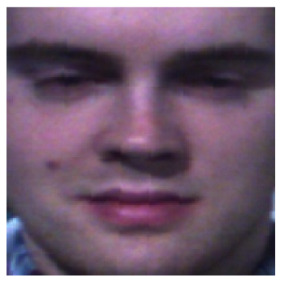	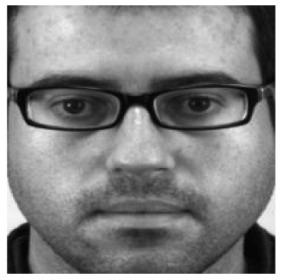	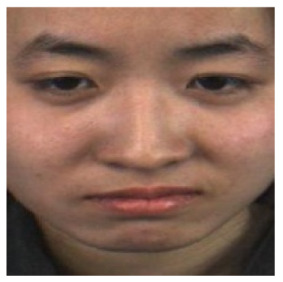
True Label	Negative	Positive	Negative	Negative
HTNet [30]	Positive	Negative	Positive	Positive
MFDA [29]	Positive	Negative	Surprise	Positive
HMRM (Ours)	Positive	Negative	Positive	Positive

## Data Availability

Our code and additional details will be available soon. The datasets that support the findings of this study are openly available at CASME II: http://casme.psych.ac.cn/casme/e2 (accessed on 15 December 2023); SMIC-HS: https://www.oulu.fi/en/university/faculties-and-units/faculty-information-technology-and-electrical-engineering/center-for-machine-vision-and-signal-analysis (accessed on 25 December 2023); SAMM: https://helward.mmu.ac.uk/STAFF/M.Yap/dataset.php (accessed on 1 December 2023).

## Abbreviations

The following abbreviations are used in this manuscript:

HMRM	Hybrid Motion and Region-Fused Mamba Network
MER	Micro-Expression Recognition
MEs	Micro-Expressions
HMFA	Hybrid Motion Feature Augmentation
GRU	Gated Recurrent Unit
GRU-AOFE	GRU-Attention Optical Flow Estimation
RFFS	Regions Feature Fusion Strategy
SSM	State Space Model
MS-WCE	Multi-Scale Weighted Cross-Entropy loss
CNN	Convolutional Neural Network
ViT	Vision Transformer
GNN	Graph Neural Network
CLBP-TOP	Completed Local Binary Patterns on Three Orthogonal Planes
LOSO	Leave-One-Subject-Out
UF1	Unweighted F1-score
UAR	Unweighted Average Recall
SOTA	State-of-The-Art
